# Variations in gene organization and DNA uptake signal sequence in the *folP *region between commensal and pathogenic *Neisseria *species

**DOI:** 10.1186/1471-2180-6-11

**Published:** 2006-02-17

**Authors:** Yvonne Qvarnstrom, Gote Swedberg

**Affiliations:** 1 Department of Medical Biochemistry and Microbiology, Uppsala University, PO Box 582, SE-751 23 Uppsala, Sweden; 2 Division of Parasitic Diseases, National Center for Infectious Diseases, Centers for Disease Control and Prevention, mail stop F36, 4770 Buford Highway, Atlanta, GA 30341, USA

## Abstract

**Background:**

Horizontal gene transfer is an important source of genetic variation among *Neisseria *species and has contributed to the spread of resistance to penicillin and sulfonamide drugs in the pathogen *Neisseria meningitidis*. Sulfonamide resistance in *Neisseria meningitidis *is mediated by altered chromosomal *folP *genes. At least some *folP *alleles conferring resistance have been horizontally acquired from other species, presumably from commensal *Neisseriae*. In this work, the DNA sequence surrounding *folP *in commensal *Neisseria *species was determined and compared to corresponding regions in pathogenic *Neisseriae*, in order to elucidate the potential for inter-species DNA transfer within this region.

**Results:**

The upstream region of *folP *displayed differences in gene order between species, including an insertion of a complete Correia element in *Neisseria lactamica *and an inversion of a larger genomic segment in *Neisseria sicca*, *Neisseria subflava *and *Neisseria mucosa*. The latter species also had DNA uptake signal sequences (DUS) in this region that were one base different from the DUS in pathogenic *Neisseriae*. Another interesting finding was evidence of a horizontal transfer event from *Neisseria lactamica *or *Neisseria cinerea *that introduced a novel *folP *allele to the meningococcal population.

**Conclusion:**

Genetic recombination events immediately upstream of *folP *and horizontal transfer have resulted in sequence differences in the *folP *region between the *Neisseria *species. This variability could be a consequence of the selective pressure on this region exerted by the use of sulfonamide drugs.

## Background

Horizontal gene transfer via natural transformation is believed to be the main mechanism to generate genetic diversity among *Neisseriae *[1-3]. Horizontal transfer has for example contributed to the spread of resistance to penicillin in pathogenic *Neisseriae*, where the origin of the transforming DNA was traced to commensal *Neisseria *species [4]. Sulfonamide resistance in some strains of *Neisseria meningitidis *(the meningococcus) has also been acquired by horizontal transfer [5]. As evidence, a mosaic pattern of fragments with high sequence divergence was detected in the chromosomal *folP *gene, the sulfonamide resistance determinant. The origin of the transforming DNA is still unknown, but presumable donors are commensal *Neisseria *species.

We have previously demonstrated that commensal *Neisseria *isolates from healthy carriers exhibit high level sulfonamide resistance, most likely due to sulfonamide-resistant variants of *folP *[6]. These commensal *folP *genes differ substantially from previously described *folP *genes, but the findings suggest that sulfonamide resistance is a property carried among species of the commensal flora. Thus, the commensal flora can be a reservoir of sulfonamide resistance genes that might be transferred to pathogenic species.

Efficient transformation is dependent upon a reasonable sequence similarity between donor and recipient to facilitate homologous recombination. In addition, DNA uptake in pathogenic *Neisseriae *is markedly enhanced by the presence of a DNA uptake signal sequence (DUS) in the donor DNA [7,8]. Thus, the sequence within and immediately surrounding *folP*, especially the presence of DUS in that region, should influence how efficient commensal *Neisseria *species may transfer sulfonamide resistance to *Neisseria meningitidis*. This work presents the gene organization and the distribution of DUS in the *folP *region in some commensal *Neisseria *species and compares these to the corresponding parts of the genomes from three *Neisseria meningitidis *strains and *Neisseria gonorrhoeae *FA1090.

## Results

### Gene organization in commensal *Neisseriae*

**Figure 1:**
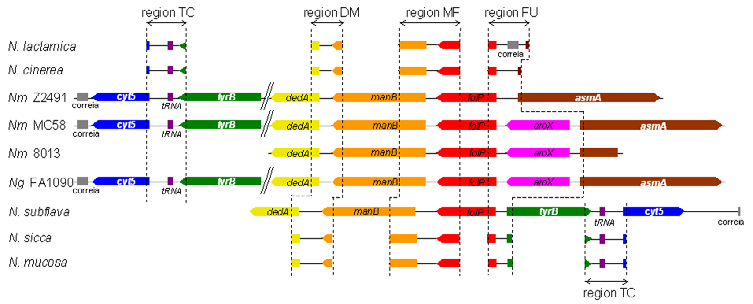
**Comparison of gene arrangements in human *Neisseriae***. The *N. subflava *sequence (accession number AJ581792 in the EMBL database) is displayed in alignment with corresponding parts from other *Neisseria *species. Open reading frames are visualized as colored block arrows (the tip of each arrow symbolizing the stop codon) and labeled with their designations according to the text. Non-coding regions are visualized as solid lines. The *dedA *gene and the Correia element in *N. subflava *were only partially sequenced. The symbol // means that 13 kb sequence separating *tyrB *from *dedA *has been omitted in the figure. The intergenic regions are defined by dotted vertical lines and designated TC (*tyrB*-*cyt5*), DM (*dedA*-*manB*), MF (*manB*-*folP*) and FU (*folP*-upstream), respectively.

The sequence surrounding *folP *was determined for *Neisseria subflava *by sequencing 6.8 kb of the genome. (*N. subflava *was chosen because sulfonamide-resistant commensal *Neisseriae *found in healthy carriers were phenotypically characterized as being most related to this species [6]). The corresponding sequences from *N. meningitidis *strains MC58 [9] and Z2491 [10], as well as *N*. *gonorrhoeae *FA1090 [11] were aligned with the *N. subflava *sequence. Included in this comparison was also a sequence from the on-going genome sequencing project of *N*. *meningitidis *strain 8013 (The Pasteur Institute, France). PCR primers designed from this alignment were then used to determine the gene order surrounding *folP *in one strain each of *N. sicca*, *N. cinerea*, *N. lactamica *and *N. mucosa*. The results are outlined in Figure 1 (see Additional file for sequence data). *N. subflava*, *N. sicca *and *N. mucosa *had identical gene order: directly downstream of *folP *was a putative phosphomanno-/phosphoglucomutase family gene (designated *manB*), followed by a putative membrane-associated gene belonging to the dedA family (*dedA*). Upstream of *folP *was an aromatic amino-acid amino-transferase gene (*tyrB*), followed by a *tRNA-Arg *gene, an open reading frame similar to a cytochrome c5 gene (*cyt5*) and a copy of the Correia repeat unit [12].

*N. gonorrhoeae*, *N. lactamica*, *N. cinerea *and the *N. meningitidis *strains had the same gene succession as *N. mucosa/N. sicca/N. subflava *downstream of *folP*, but the genes found upstream of *folP *were completely different. Instead of being located upstream *folP*, the gene sequence *tyrB*-*tRNA*-*cyt5*-Correia was in these species located 14 kb downstream of *folP*. The direction of *tyrB *and the following genes in relation to *folP *was also the opposite compared to the case in *N. subflava/N. sicca/N. mucosa*. This indicates that the difference in gene organization was due to an inversion of a genomic segment consisting of the *folP *gene and the region between *folP *and *tyrB*. Another interesting observation was that the distance between *folP *and the upstream gene in *N. lactamica *was longer than in other species, due to the insertion of a Correia element. This Correia element was identical to some of the elements found in pathogenic *Neisseriae*. It had the typical features for these repeats: it was flanked by TA repeats, had a putative integration host factor binding site and its length and inverted terminal repeats indicated that it belonged to the α family of Correia elements [13].

### Presence of the *aroX *gene

**Figure 2:**
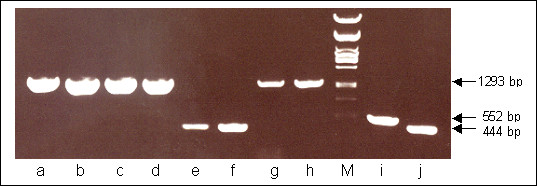
**PCR amplification of the region between *folP *and *asmA *in *Neisseriae***. A 1293 base pair product denotes the presence of *aroX *between *folP *and *asmA*. M: molecular marker; a: *Neisseria meningitidis *(Nm) strain 952 (Su^S^); b: Nm 1014 (Su^S^); c: Nm BT054 (Su^S^); d: Nm 418 (Su^R^); e: Nm 3976 (Su^R^); f: Nm BT227 (Su^R^); g: Nm MO035 (Su^R^); h: Nm MO124 (Su^R^); i: *Neisseria lactamica *(Su^S^); j: *Neisseria cinerea *(Su^S^). Su^S ^= sulfonamide-susceptible, Su^R ^= sulfonamide-resistant.

Two of the meningococcal strains (MC58 and 8013) and *N. gonorrhoeae *FA1090 had a putative chorismate mutase-related gene (*aroX*) immediately upstream of *folP *followed by a putative *asmA *gene (see Figure 1). The third meningococcal strain (Z2491), *N lactamica *and *N. cinerea *lacked *aroX *in this position and instead had *asmA *next to *folP*. PCR analysis revealed that *aroX *was present upstream of *folP *in six out of eight clinical strains examined (see Figure 2). Interestingly, the two clinical strains lacking *aroX *both had *folP *genes identical to Z2491. This *folP *allele is associated with sulfonamide resistance [5] and displays a very high degree of sequence divergence surrounding the start codon of the gene, compared to other meningococci: 26 altered positions in a 61 base pairs sequence. In fact, this divergent part was almost identical to corresponding sequences in *N. cinerea *and *N. lactamica*, suggesting that horizontal transfer from one of these commensal species removed the *aroX *gene and simultaneously altered the "wild type" meningococcal sequence immediately upstream of, and the first part of, *folP*.

### Difference in DNA uptake signal sequences

**Figure 3:**
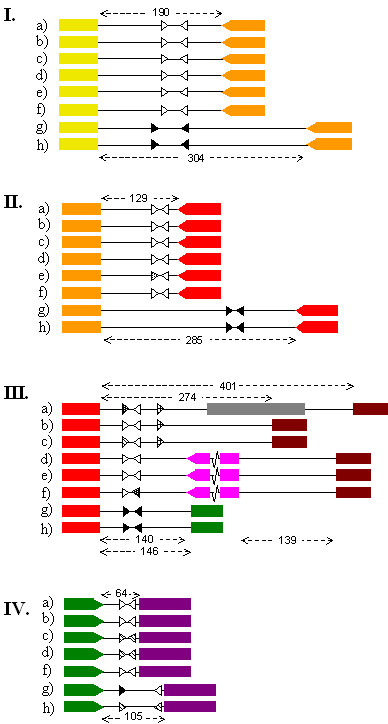
**Enlargement of the intergenic regions from Figure 1**. I: region DM. II: region MF. III: region FU. IV: part of region TC. The order of the strains is as in Figure 1: a) *Neisseria lactamica*; b) *Neisseria cinerea*; c) *N. meningitidis *Z2491; d) *N. meningitidis *MC58; e) *N. meningitidis *8013; f) *N. gonorrhoeae*; g) *Neisseria subflava*; h) *Neisseria sicca*. Numbers flanked by dotted arrows specify distances in base pairs between adjacent start- and stop codons. Triangles illustrate various DNA uptake signal sequences: ▷: GCCGTCTGAA; ▶: G**T**CGTCTGAA; : GCCGT**T**TGAA; : GCCGTC**C**GAA; : GCCGT**A**TGAA; : **A**CCGTCTGAA; : **AT**CGTCTGAA. Triangles pointing towards each other illustrate inverted repeats. For the complete nucleotide sequences, see Additional file.

The complete 6.8 kb sequence obtained from *N. subflava *and the intergenic regions amplified by PCR from *N. sicca*, *N. lactamica *and *N. cinerea *were examined for presence of the neisserial DNA uptake signal sequence (DUS) 5'-GCCGTCTGAA-3'. The meningococcal and gonococcal genomes contain almost 2000 copies of this sequence, which makes an average of one copy every 1,100 base pairs. The intergenic sequences from *N. lactamica *and *N. cinerea *were highly similar to the corresponding regions from the pathogenic species, including the numbers and positions of DUS (see Figure 3). In contrast, only one copy of the neisserial DUS was found in the *N. subflava *sequence and none was found among the *N. sicca *intergenic sequences. However, an altered variant where the second base is a thymine (5'-GTCGTCTGAA-3') was more prevalent in the latter species: *N. subflava *displayed as many as 10 copies of this altered DUS (see Figure 3; three of the altered DUS are located outside of the displayed sequences) and 6 copies was found in *N. sicca*. The occurrence of the neisserial and the altered DUS in the *folP *region of various *Neisseria *species is listed in the left part of Table 2.

To extend the analysis beyond the *folP *region, all sequences from human *Neisseria *species that were larger than 400 base pairs and deposited in the GenBank as of October 2005, were screened for both types of DUS described above. As a comparison, we also searched for another variant of DUS, where the third nucleotide was a thymine (5'-GCTGTCTGAA-3'). The results are summarized in the right part of Table 2. The altered DUS 5'-GTCGTCTGAA-3' was detected in the pathogenic *Neisseria *genomes and in sequences from *N. cinerea, N. subflava*, *N. mucosa *and *N. elongata*, but not as frequent as found around *folP *in *N. subflava *and *N. sicca*. Furthermore, all species also had several copies of the neisserial DUS, present at a much higher density than the altered variant. This indicates that the neisserial DUS is the prevalent one in all *Neisseria *species, and contradicts our finding from the *folP *region in *N. subflava *and *N. sicca*. When looking at this region in other species, several different variants of DUS can be identified (see Figure 3). For example, the region upstream of *folP *displays four variants of DUS: the neisserial DUS, the altered DUS and two other variants. The last column in Table 2 lists the prevalence of a DUS with an altered third base: this variant was also found in most *Neisseria *species. Obviously, the genomes of *Neisseria *species contain multiple variants of DUS, which seem to be scattered in variable densities throughout the genomes.

### Transformation with DNA containing altered DUS

The transformation efficiency for DNA containing the neisserial DUS (5'-GCCGTCTGAA-3') and the altered DUS (5'-GTCGTCTGAA-3') was determined for *N. meningitidis *strain 952. Transformation frequencies were highest for DNA containing the neisserial DUS and around 50% lower for DNA with the altered DUS. However, the absolute transformation frequencies varied between experiments, so there was no statistically significant difference between the two DUS variants (see Table 3). DNA with a distorted DUS variant, where four bases had been replaced, scored significantly lower values, indicating that the DNA uptake was indeed sequence dependent, as expected in *N. meningitidis*. The conclusion from these experiments was therefore that a one-base-substitution in the DUS did not produce a significant reduction in transformation frequency in this laboratory setting.

## Discussion

The *Neisseria *species are believed to be non-clonal bacteria with a high degree of genetic transfer within and between different species [2,3,14-18]. Previous reports of transformation in *N. meningitidis *have suggested that different regions of the genome may have different recombination rates [19,20]. This work identified the region upstream of *folP *as particularly active in recombination events. The most obvious example is the insertion of a complete Correia element, upstream of *folP *in *N. lactamica*. Correia elements are small putative transposable elements, around 152 base pairs long with inverted repeats at the ends [12]. Hundreds of Correia copies are scattered around the genomes of pathogenic *Neisseriae *[13,21,22] and they have also been reported in *N. lactamica *[23]. We detected another copy of a Correia repeat in the *N. subflava *sequence, suggesting that these elements are ubiquitous in the *Neisseria *genus and possibly was introduced before the divergence into different species.

Another example of recombination events is the apparent inversion of a genomic segment in *N. subflava*, *N. sicca *and *N. mucosa*, with one breakpoint upstream of *folP*. A third recombination event in the same location is the deletion of *aroX *from some strains. This gene is obviously not essential since it is absent from the genome of *N. meningitidis *Z2491, but was nevertheless present in the majority of strains examined in this work. The sequences between *folP *and *aroX *from all these latter strains are highly similar and include two DUS downstream of *aroX*, arranged as an inverted pair followed by a stretch of thymidines. This arrangement is characteristic of transcription terminators and is a general configuration for DUS in meningococcal and gonococcal genomes [8,24]. The strains lacking *aroX *had retained this putative termination signal, although located between two divergently operating start codons and hence without function. Furthermore, all commensal strains examined in this work also had inverted repeated DUS between the start codons of *folP *and the upstream gene (*tyrB *or *asmA*). These observations support the proposed genetic rearrangements in these species: a deletion of *aroX *in *N. lactamica*, *N. cinerea *and some meningococcal strains, and an inversion of a genomic segment in *N. subflava*, *N. mucosa *and *N. sicca*.

The sequence differences and similarities detected in this work could divide the examined species into two groups, where the pathogenic species, *N. lactamica *and *N*. *cinerea *fall in one group and *N. subflava*, *N. sicca *and *N. mucosa *in a second group. This is in agreement with the classic taxonomy of *Neisseria *species based on ribosomal RNA hybridization [25,26] and phenotypic tests [27]. The inversion of the *folP*-*tyrB *genomic fragment and the accumulation of the altered DUS likely occurred in a common ancestor to *N. subflava*,*N. sicca *and *N. mucosa*. Similarly, the deletion of *aroX *probably occurred in a common ancestor to *N. lactamica *and *N. cinerea*, but after the separation of commensal species from pathogenic species. The removal of *aroX *created a novel intergenic sequence that was later transferred to a meningococcus, removing *aroX *also in this strain. The fact that all meningococcal strains we found lacking *aroX *were sulfonamide-resistant and had identical *folP *genes can be explained in three ways: either, the recipient was already sulfonamide-resistant at the time of transfer; the recipient acquired resistance after the transfer; or the donor was a sulfonamide-resistant commensal strain that transferred the resistant *folP *allele together with the upstream region to the meningococcus.

The DNA uptake signal sequence (DUS) is crucial for efficient transformation in pathogenic *Neisseriae *[7,8]. The tendency to preferentially take up DNA from closely related species was noted in early experiments with gonococci [28-31]. Further experiments established that this was due to the presence of an uptake signal sequence in the donor DNA, and this sequence was subsequently determined to be 5'GCCGTCTGAA-3' [7,8,32]. The meningococcal and gonococcal genomes contain close to 2,000 copies of this neisserial DUS, but they also contain several variants of this sequence with one or a few base substitutions. The altered variants of DUS are frequently located in pairs as perfect inverted repeats after open reading frames, just as the neisserial DUS. This could indicate that this arrangement is important for its function and therefore support the hypothesis of DUS as being transcription terminators [24]. The DUS variant with a thymine in second position was highly prevalent in the *folP *region of *N. subflava *and *N. sicca*, where it seemingly had replaced the neisserial DUS, and was also fairly abundant in pathogenic strains: 165 and 118 copies in *N. meningitidis *MC58 and *N. gonorrhoeae *FA1090, respectively. The results from our transformation experiments, albeit performed with synthetic PCR-products under conditions different from the natural habitat of these bacteria, indicated that this one-base difference in DUS only marginally reduced the transformation frequency. Perhaps this can explain why this particular variant of DUS seems so prevalent in neisserial genomes: this alteration may be well tolerated by the DNA uptake mechanism.

It is not clear why some naturally competent species have a sequence-specific DNA uptake mechanism. One explanation describes their presence as a mere consequence of a preferential uptake of specific sequences during natural transformation [33]: DNA uptake processes that favor a specific sequence automatically lead to an accumulation of this preferred sequence in the genome, because DNA containing this sequence is more likely to be integrated than other DNA. Another theory is that DUS may be beneficial for the bacterial population since it directs the DNA uptake to sequences from closely related bacteria that can be used for creation of genetic variability or for DNA repair. A recent study report a higher frequency of DUS in genome maintenance genes compared to genes of other function, thus supporting the DNA repair explanation [34]. If DUS evolved as a mechanism to obtain homologous DNA for repair purposes it would make sense that only closely related species shared the same DUS. This hypothesis could therefore justify why the neisserial DUS was found in *N. lactamica *and *N. cinerea*, whereas an altered version of DUS was detected in the more distantly related *N. subflava *and *N. sicca*. On the other hand, this distinction was only true for the *folP *region: when examining sequences from other regions the neisserial DUS dominated in all species. However, the sequences available for analysis from several of the commensal species, including *N. subflava *and *N. sicca*, are in short supply and not representative for the whole genomes. Obviously, analysis of more sequences from these commensals is required to address the distribution of various DUS within the genomes of commensal *Neisseria *species.

## Conclusion

This work compared the sequence surrounding the *folP *gene in various *Neisseria *species. The results lead to a general differentiation of the examined species into two groups, where *N. subflava*, *N. mucosa *and *N. sicca *belonged to one group and *N. cinerea*, *N. lactamica*, *N. meningitidis *and *N. gonorrhoeae *to another group. The major differences between these two groups were an inversion of DNA with one breakpoint in the upstream region of *folP*, and a one base pair difference in the DNA uptake signal sequences present in this genomic region. The variability within the *folP *region may reflect the ongoing selection of different *folP *alleles by the selective pressure of sulfonamide drug use.

## Methods

### *Neisseria *strains

The *Neisseria meningitidis *clinical isolates used in this work were 418, 1014 [35], MO035 [36], 952, BT054, BT227, MO124 [37] and 3976 [38]. The commensal strains were *Neisseria lactamica *ATCC23970, *Neisseria mucosa *ATCC19696, *Neisseria sicca *ATCC9913, *Neisseria subflava *ATCC19243 and *Neisseria cinerea *ATCC14685. Bacteria were cultivated on Brain Heart Infusion agar plates (Difco Laboratories, USA) in 10% CO_2_.

### Reagents

Oligonucleotides (listed in Table 1) were purchased from Thermo Hybaid, Germany. Restriction enzymes and other molecular biology reagents were purchased from Roche Diagnostics, Germany, if not otherwise stated. Ligation mixtures were transformed into One Shot^® ^TOP10 Chemically Competent *E. coli *(Invitrogen™, The Netherlands). PCR products were purified from agarose gels using MinElute Gel Extraction Kit (QIAGEN, Germany) and cloned into pCR^®^2.1-TOPO^® ^vector (Invitrogen™, The Netherlands) according to the manufacturer's instructions. Plasmids were purified using QIAprep Spin Miniprep (QIAGEN, Germany). DNA was sequenced on ABI PRISM 310 Genetic Analyzer (Applied Biosystems, USA) using DYEnamic ET Terminator Cycle Sequencing Kit (Amersham Biosciences, Sweden).

### Determination of *Neisseria subflava *sequence

PCR primers NM7 and NM11 were used to amplify 734 base pairs of *folP *from *N. subflava *ATCC19243. Flanking sequences were determined using a chromosome walking method [39]; Restriction enzyme digested chromosomal DNA (approximately 2 μg) was inserted into vector pUC19 with T4 DNA Ligase (New England Biolabs, Beverly, MA) at 16°C. The unknown sequence was then amplified with the ligation mixture as template and one vector-specific primer, directed towards the insert, and a sequence-specific primer, directed towards the unknown sequence. The polymerase chain reactions contained 0.2 units Taq Polymerase (Fermentas, Lithuania), 0.5 μM of each primer, 0.2 mM of each deoxynucleotide and 100 ng of DNA in a 20-μl reaction. PCR products were cloned and sequenced as described above. The chromosome walking was repeated stepwise in both directions until a 6808 base pair region of the chromosome had been sequenced. Nested PCR was performed in some of the steps to achieve specific products.

### Determination of gene order

Primers binding within genes but directed towards non-coding regions were utilized to amplify intergenic sequences from chromosomal DNA from all *Neisseria *strains listed above using the Expand™ High Fidelity PCR System (Roche, Germany). Primers dedAup and Nsfmutasdown3 were used to amplify the region between *dedA *and *manB*; Nsfmutasrev5 and NM10 to amplify the region between *manB *and *folP*; Nsfupanti and NmAsmAup between *folP *and *asmA*; Nsfupanti and NsftyrBup between *folP *and *tyrB*; and NsftyrBdown2 and cytup between *tyrB *and *cyt5*. PCR products from *N. lactamica*, *N. cinerea *and *N. sicca *were cloned and sequenced as described above.

### Transformation frequencies

The complete *folP *gene from the sulfonamide-resistant *N. meningitidis *strain MO035 was amplified from chromosomal DNA using the forward primer Nmdusfwd and one of three reverse primers. NmdusCrev was used to incorporate the meningococcal DUS in the PCR-product, NmdusTrev to incorporate the subflava variant of DUS and NmdusXrev to make a control DNA with equal length but with a disrupted DUS. PCR was performed with the Expand™ High Fidelity PCR System (Roche, Germany) according to the manufacturer's instructions and the products were purified with the MinElute PCR Purification kit (QIAGEN, Germany). The sulfonamide-susceptible *N. meningitidis *strain 952 was suspended to OD_600 _= 0.1 in a rich broth containing Brain-Heart-Infusion Blood-Agar Base (Difco Laboratories, USA), 10 mM MgCl_2_, 2× Kellogg's supplement and 0.042% NaHCO_3 _[40]. Exactly 1 μg of purified PCR-products was added to 200 μl bacterial suspension and the mixture was incubated at 37°C in 5% CO_2 _until OD_600 _= 0.3. The diluted transformation mixtures were incubated on ISA plates (Oxoid, England) with or without 0.25 mM sulfathiazole (Sigma-Aldrich, Sweden).

### Sequence data analysis

The complete genome sequences from pathogenic *Neisseria *species were extracted from GenBank: AL157959 (strain Z2491), NC003112 (MC58) and AF00969 (FA1090). The 5 kb sequence from *N. meningitidis *strain 8013 was provided by Christophe Rusniok and Philippe Glaser at the Pasteur Institute, France. The following entries from commensal *Neisseria *species were extracted from GenBank: *N. cinerea*: AF158604, Z17308-Z17310, X64869, AJ223935, AJ223913-AJ223915, AJ223890-AJ223892, AY831725, AJ704731, AY360352, AF470674, AF319536, AF306836, AF139614, AF216744, AJ247236, AJ239300, AJ239299, AJ239287, AF121876, AF029360, U82861, U82845, AF85340, AJ223869-AJ223871, U57906, U57710, X59540; *N. elongata*: AF121870, DQ007933, AJ223877-AJ223882, AJ223921-AJ223926, AJ223898-AJ223903, AY821559, AY167422, AF542173, AJ247252-AJ247254, AJ239302, AJ239303, AJ239297, AJ239278, AF121870, L06171; *N. flavescens*: M26645, AY174058, Z22955, X64872, AJ223938, AJ704736, AJ79434, AJ704735, AJ704733, AJ239280, U82863, U82847, AF987677, Y09310, Y09311, U57907, U57711, L06168; *N. lactamica*: DQ015867, Y11818, Y11819, Y10876, Y10877, X54485, X64871, AJ288888-AJ288893, AJ223909, AJ223945-AJ223947, AJ223910, AJ223907, AJ223908, AJ223884-AJ223887, AJ223863-AJ223866, NC006968, AY857302-AY857306, AY532623-AY532628, AY532630, AY54849, AY654848, AJ704746-AJ704748, AJ704737, AY438701, Y134876, AF542177, AJ270921-AJ270932, AJ270918, AJ270906-AJ270908, AJ270902, AJ270900-AJ270904, AF470682, AF470683, AF470673, AY038932, AF319537, AJ270919, AJ270920, AF312972, AF216855, AF158603, AJ247241, AJ247242, AJ239313, AJ239305, AJ239296, AJ239283-AJ239286, U82843, U82858, AF085689, AJ001737-AJ001740, Y09308, U57709, Y57905, U59398, X74901, X65533, U06074; *N. mucosa*: AJ223875, X59635, X64873, AJ223937, AJ223919, AJ223896, AJ223875, AJ704738, AY462277, AJ247255-AJ247260, AJ239279, AJ239282, AF121872, U82860, U82848, L47156, U57908, U57901; *N. perflava*: AJ223920, AJ223906, AJ223897, AJ223883, AJ223876, AJ223862, AY837569, AF312973, AJ247246, AJ247247, AJ239295; *N. polysaccharea*: DQ007935, Y11814-Y11817, Y10878-Y10881, X59626, X64870, AJ223944, AJ223940-AJ223943, AJ704761, AJ704762, AJ704743, AJ704739-AJ704744, AY134878, AY072807-AY072809, AY099335, AF542178, AF216858, AJ239314, AJ239315, AJ239298, AJ239289, AJ011781, U82844, U82859, Y09309, U57708, U57904, L06167; *N. sicca*: DQ007936, X76285, AJ223916, AJ223872, AJ223893, AY857301, AJ704730, AF312974, AJ247248, AJ239292-AJ239294, Y09307; *N. subflava*: DQ007937, AJ288894, AJ223918, AJ223895, AJ223874, AY522860, AJ704745, AY134877, AF216745, AF240672, AF241526, AJ247249, AF470684, AF479577, AF479578, AJ239291. The search for DUS and Correia elements was performed using the DNA Pattern Find algorithm in the Sequence Manipulation Suite [41].

## Authors' contributions

YQ participated in the planning of this study, performed all experimental work and drafted the manuscript. GS participated in the planning, coordinated the study, and assisted in writing the manuscript. Both authors have approved the final manuscript.

## Supplementary Material

Additional File 1 **Intergenic sequences from commensal *Neisseriae***. This document lists the nucleotide sequences from the commensal species that are schematically illustrated in Figure 3. Click here for file

## Figures and Tables

**Table 1:** Oligonucleotides used as PCR primers.

**name**	**sequence (reference)**
cytup	5'-TAACGATACCGCTCACAAGGG-3'
dedAup	5'-GTCAAATCTTCGGGAATCGG-3'
NM10	[42]
NM11	CCGCCGCCACGCTGCCGTGT
NM7	TTGGCAGGCAGGACGGTTTG
NmAsmup	5'-CATATTTCAGACGGTATTTGTGG-3'
NmdusCrev	CCTTTTCAGACGGCATGAAGTATGTGAACC
Nmdusfwd	GTTTTTTATTTTGGGAGGAGTTC
NmdusTrev	CCTTTTCAGACGACATGAAGTATGTGAACC
NmdusXrev	CCTTTTGTGAGTGCATGAAGTATGTGAACC
Nsfcytokrom1	5'-TATCGCGCAAGGCTTCGATAC-3'
Nsfcytokrom2	5'-TAACGCCATGCCTGCAAAAG-3'
Nsfcytokrom3	5'-AGAAACCCTGCACAAACACG-3
Nsfcytokrom5	5'-AACGCCATGCCTGCAAAAG-3'
Nsfdownsense3	5'-CAGGTTTCCCGCTGTTGATCG-3'
Nsfmutasdown2	5'-TGCGCATCCAAAAAGGTCAG-3'
Nsfmutasdown3	5'-AAAGACGTGTTGGCGGAAGTG-3'
Nsfmutasdown4	5'-GGGAACGGTGGTACAGTGGAT-3'
Nsfmutasdown5	5'-CCATGCCGGTTTAAGGTTAAT-3'
Nsfmutasfwd6	TATTCCGACAACGGCATCAAATTC
Nsfmutasfwd7	5'-GTGAAGCTGAGCGATGAAATCGAG-3'
Nsfmutasrev5	5'-GTCGATTTTGGCTTCGATTTCC-3'
NsftyrBdown1	5'-GATTGAGCGTGGAACAAGTCCG-3'
NsftyrBdown2	5'-ATTCCGGCAGGATGTGCGTC-3'
NsftyrBfwd1	5'-ACCGGCGTGGATATGTCGG-3'
NsftyrBup	5'-GGTCGTTTTTGAAGGTTTCGAC-3'
Nsfupanti	5'-GTTTGGGCGTTTTGCGAATA-3'

**Table 2:** Presence of DNA uptake signal sequences in human *Neisseria *species.

	**Prevalence (density^1^) of DNA uptake signal sequences**					
	**in intergenic parts of the *folP *region (Fig. 3)**		**in database sequences**			
	
**Species**	GCCGTCTGAA	G**T**CGTCTGAA	**Sequence length**	GCCGTCTGAA	G**T**CGTCTGAA	GC**T**GTCTGAA

*N. meningitidis*^2^	6	0	2,272,300	1935 (1,174)	165 (13,772)	47 (48,347)
*N. gonorrhoeae*	7	0	2,153,894	1965 (1,096)	118 (18,253)	31 (69,480)
*N. lactamica*	7	0	51,533	40 (1,288)	0	0
*N. cinerea*	8	0	12,158	15 (811)	3 (4,053)	4 (3,040)
*N. subflava*	1	7	13,523	16 (845)	1 (13,523)	0
*N. sicca*	0	6	3,114	3	0	1
*N. mucosa*	n.a	n.a	4,492	3	1	1
*N. polysaccharea*	n.a	n.a.	22,903	25 (916)	0	2 (11,452)
*N. flavescens*	n.a	n.a	4,844	8	0	0
*N. perflava*	n.a	n.a	1,392	2	0	0
*N. elongata*	n.a	n.a	7,064	8	1	0

^1 ^Density as the average distance between two DUS. Density was only calculated when the (accumulated) sequences included more than 10,000 base pairs.

^2 ^Strain MC58

**Table 3:** Transformation frequencies

**Type of DUS**	**Average transformation frequency per 10,000 cells **(mean ± SD)
neisserial DUS (GCCGTCTGAA)	56 ± 21
altered DUS (G**T**CGTCTAA)	27 ± 15
distorted DUS (CG**AC**TC**AC**AA)	4 ± 2
