# Supplementary material for: Variations in gene organization and DNA uptake signal sequence in the folP region between commensal and pathogenic Neisseria species
Source: BMC Microbiol. 2006 Feb 17;6:11. doi: 10.1186/1471-2180-6-11 (PMC1431543; doi:10.1186/1471-2180-6-11)
Supplement: Additional File 1 — Intergenic sequences from commensal Neisseriae. This document lists the nucleotide sequences from the commensal species that are schematically illustrated in Figure 3. [file 1471-2180-6-11-S1.htm]

SUPPLEMENTARY INFORMATION


# SUPPLEMENTARY INFORMATION

 

Manuscript:

Y. Qvarnstrom and G. Swedberg: Variations
in gene organisation and DNA uptake signal sequence in the *folP* region
between commensal and pathogenic *Neisseria* species.

 

# Intergenic sequences from commensal *Neisseriae*.

# 

# dedA-manB (region DM)

 

### N. cinerea ATCC14685

CACGCCGAAACCGCAGATGACCAATACAAAAAAAACAGCCGCATAACCGTATTCGACAAAAAAGGCTTCTAAAAAAGCAAACATGGCGGATATTCCATTGTCGGAGATAAAAAGTCAGAACAAACCGAAACATTTTCTACATGAAACAGCATTCTATCAAAGATTATGCCGTCTGAAAGCGGAAAAAAGGCAGATTCGCGCATCCTCCGCATGTTCAGACGGCATTTAAACAGAAAACCGGCCGGTTCTGAAAACGGTTTTTCCTGATTTTGCCTAAATGCTGCCGATGGCGGAAGCAATACGTTCCGCCCCTTCGCGCGCCCAATCGGCCTGTCGTGCCTCAACCATCACGCGCACGACGGGTTCAGTTCCCGAAGCGCGCAACACAACCCTTCCTTTGCCTTCGAGTTCTTTTTC

 

**N. lactamica ATCC23970**

CACGCCGAAACCGCAGATGACCAAAACGAAAAACACGGCTGCGTAGCCGTATTCGACAAAAAAGGCTTCCAAAAGGGCAAACATAGCGGGTTTTTCCATTGGTCGGAAATAAAAAGTCAGAACAAGCCGAGGCGTTTTCTGCGCGAAACGGCATTCTATCAAAGATTATGCCGTCTGAAAGCGGCAGGGGGCAGATTCGCGTGCCCGCCGCCCGTTCAGACGGCATTTAAACAGAAAACCGCCCGGTTCTGAAAACGGTTTTGCCTGATTTTGCCTAAATGCCGCCGATGGCGGCGGCGATACGTTCCGCCCCGTCGCGCGCCCAATCCGCCTGCCGCGCTTCAACCATCACGCGCACGACCGGTTCGGTTCCCGAAGCGCGCAACACAACCCTTCCTTTGCCTTCGAGTTCTTTTTC

 

### N. sicca ATCC9913

TACGCCAAAGCCGCAGATGACCAAAACGAAAAACACGGCTGCGTAGCCGTATTGGACAAAAAAGGCTTCTAAAAAGGCAAACATGGCGTATCCGTCGGTTGTAATGTGGAGGGGAAATTCATTTGAACTGAATGTCGTATTTTACCAAAGATATGGTCGTCTGAAAGAAATAACAAGGAAGGTTATGAAAATAGGTGTGAACAATATGAATTGATGTGTTTCAGACGACCTCGAATCCTAAAAAACACAGTGGATTAACCTTAAACCGGCATGGCGTTCCTCCACCTTGCCCTGATTCAAAATTAATCCACTGTACCGCCGTTCCCGCTTGCGTGGAAACGACGGCAGTTCCAAGGTTTGCTAAAAACCTATTGATTTTTCGACGGCATTACTGTTTGCCGGTGATGGCTGCTGCGATGCGTTCCGCGCCTTTTTTCGCCCAGTCCGCCTGACGCGCTTCTACCATGACGCGCACGACCGGCTCGGTACCCGATGCGCGCAACACGACGCGGCCTTTGCCTTCGAGTTCTTTTTC

**manB -folP (region MF)**

**N. lactamica ATCC23970**

```
AACTCGACTTCATCGCTCAATTTCACGCCGCCCTCGGCAAAGAATTTGATGCCGTTGTCGGAATAGGCATTGTGCGACGCGGAAATCATCACACCGTCCGGACAAACGCAGCGCACGGGTCAGATAAGCCACGCCGGGCCGTAGGCAGCGGACCGGTTTGGATAACGTTGACACCTGCGGCGGTAAAACCCGCCACCAAAGCGGCCTCCAGCATATAGCCGGAAATCCGCGTATCCTTGCCGATTAAAACAGTCGGTTTCTGGCCGGTGTCGTGCTGAACCAAAACCTGCCCCGCCGCATAGCCGAGTTTAATACAAAATCGGGCGTAATCGGAAACTGCCCGACTTCGCCGCGCACGCCGTCCGTGCCGAAATATTTTTTTGCCATCATTTGCTCCGAGCATGTGAACCGTTGTCCGAGATTATACAGGCAGTTTGTGCCTTGCTGTCTGCACCATTGATGCCGTCTGAAGCCGCCCcGCCCTTTTCAGACGGCATAAAGTATGCGAACCGCTGTTTACAGATTGATGCCCAACGCTTCCCACACCTTCAACGCATCCGCCGTCGCCTTCACATCATGCACCCGCACGATTTGCGCGCCGCGCGCCACGGAAGCCAGCGCCGCCGCCACGCTGCCGTGTACGCgTgcCGCCGCGTCTGCCTCGCCGGTCAGCTCGCCTATCATGCGCTTGCGCGACACGCCGACCAGCAGCGGCAAAcCGGTTTCCGCCATCAATTCGGGCAAATGCCGcATCAGCGCGATATTGTGTTGCAGGGTTTTGCCGAAGCCGAAACCCGGGTCGAGTGTGATG
```

### 

 

### N. cinerea ATCC14685

```
AACTCGATTTCATCGGAAAGCTTCACGCCACCCTCGGCAAAGAATTTGATGCCGTTGTCGGAATAGGCATTGTGCGACGCGGAAATCATCACACCGGCGGATAAACGCAGCGCACGGGTCAGATAAGCCACGCCGGGCGTAGGCAGCGGACCGGTTTGGATAACGTTGACACCTGCGGCGGTAAAACCTGCCACCAAAGCGGCTTCCAGCATATAACCGGAAATACGCGTATCCTTGCCGATTAAAACGGTCGGTTTCTGGTCAGTGTCGTGCTGAACCAAAACCTGACCCGCCGCATAGCCGAGTTTAATACAAAATCGGGCGTAATCGGAAACTGCCCGACTTCGCCGCGCACGCCGTCCGTGCCGAAATATTTTTTTGCCATGTGTTGCTCCGAGAATGTGAACCGTTGTCCGAGATTATACAGTCAGTTTGTGCCTTGCTGTCTGCACCGTTGATGCCGTCTGAAGCCGCCCCGTCCTTTTCAGACGGCATGAAGTATGTGAACCGCTGTTTACAGATTGATGCCCAACGCTTCCCACGCCTTCAAAGCATCCGCCGTCGCCTTCACATCATGCACCCGCACAATTTGCGCGCCGCGCGCCACGGAAGCCAGCGCCGCCGCCACGCTGCCGTGTACGCGTGCCGCCGCGTCTGCCTCACCGGTCAGCTCGCCTATCATGCGTTTGCGCGATACGCCGATTAGCAATGGCAAACCGGTTTCCGCCATCAATTCGGGTAAATGCCGCATCAGTGCGATATTGTGTTGTAAGGTTTTGCCGAAGCCGAAACGGGGTCGAGTGTGATG
```

### 

### N. sicca ATCC9913

```
AACTCGATTTCNTCGCTCAGTTTCGCCGCCTTCGGCGAAGAATTTGATGCCGTTGTCGGAATATACATTGTGCGACGCGGAAATCATCACGCCGGCGGACAAACGCAGCGCACGGGTCAGATAAGCCACGCCCGGCGTAGGCAGCGGGCCGGTTTGGATGACGTTGACACCCGCGGCGGTAAAACCCGCCACCAGCGCGGCTTCAAGCATGTAGCCGGAAATACGCGTGTCTTTGCCGATAAGGACGGTCGGTTTCTGCCCGCCGTCATGTTGCACCAACACCTGCCCCGCCGCATAACCGAGTTTCAATACGAAATCGGGGGTAATCGGGAATTGACCGACTTCGCCGCGCACGCCGTCTGTGCCGAAATATTTTTTTGCCATGAGTTTACTCCGTGAGGATGTTTAGAATTGGGGTTACCGTAAATGTCATAACGGGCAGTATAGGGAATTTTATGCAAAACTGTTGGGTTTTATGTTTAGCGTTATTAGATTGCGCCTGAGCGGTATAGTTTCTTATCTTATCAAAACCCGAAACAAGTTAACCGGATTAAACAAAAGGGTCGTCTGAAACCGCCAAACTCGTTTTCAGACGACCTCTTTATCACTATTTCAGGGATTGCCGACATTTCATTTTCAAAGTGTTTTTTGTTTGCAAAGCAGAAGCTATTAAATGCCGGTAATGCCTAAGGCGTCCCACACCTTCAACGCATCCGCCGTCGCTTTCACATCATGCACCCGCACAATTTGCGCGCCGCGCGCTACGGACGCCAACGCCGCCGCCACGCTGCCGTGAACACGTTCCGCCGCGTCTGTCTCGCCGGTCAGCTCGCCTATCATGCGTTTGCGCGACACGCCGATGAGCAGCGGAAAACCCGTTTCCGCCATCAATTCGGGCAAATGCCGCATCAGCGCGATATTGTGTTGCAGGGTTTTGCCGAAGCCAAAACCGGGGTCAAGCACGATG
```

 

**folP - upstream (region FU)**

 

### N. sicca ATCC9913

```
CACGCCGCCATCGGAAAACGAATCGGGCGTCAAGTTGACGATACCCATGATTTTCGGTTTGTCCAAACCAATTTCAAACCTGCCTACCTGCCAGATGCGCGTGGTCATGATGGTGTTCCGTAAAATAATGAAGACGAAATTATAGGTCGTCTGAAAACATCATGCAGCTTTCAGACGACCCCGTTTTACAAATCCTGCCGTTTAATGCCAAAATCAGGTTTACCGTCCTAATCCGCCTGACAAAGGAAGCATCATGTACCAACACGTCGAATACTACCCCGGCGACCCGATTTTGAGTTTG
```

 

### N. cinerea ATCC14685

CGCGCCGCCGTCGGAAAAAGAATCGGGCGTGAGATTCACGATACCCATGATTTTCGGTTTGTCCAAACCGATTTCAAACCGTCCTGCCTGCCAAACGCATCCGACCATATCCGTGCCTCCGGAATAAAAAGCAAATTATATGCCGTCTGAACAAAACTTGTCCGTTTCAGACGGCATCGGTATTTCAAAAAAGTATCTTGCAGAATCCGCCGCCGTATGAAACATACACTGCCCCTGCAAACGCGGCAACACCCGCGATACCTGATCCGGCGGTATGCCTGCTTATGGACATTACCCCTTTATACAAGATAATCCGCCCGCACAAAATAGGATTTCTACAAGCCGTGTTATACTGTGGCGTGTTTTACAGACTTTTCAGGCTATGGATTTATTATCGGTCTTCCACAAATACCGTCTGAAATATG

 

**N. lactamica ATCC23970**

```
CGCGCCGCCGTCGGAAAAAGAATCGGGCGTGAGATTCACGATGCCCATGATTTTCGGCTTGTCCAAACCGATTTCAAACCGTCCTGCCTGCCAAACGCATCCGACCATATCCGTGCCTCCGGAATAAAAAGCAAATTATATGCCGTCCGAACAAAACTTGTCCGTTTCAGACGGCATCGGCATTTCAAAAAAGCCTCCCGCCAACTCCGCCGCCGTATGAAATATGCCcGCCCCTGCGAACGCGGCAACACCCCTTTATGCAAGATATAGTGGATTAACAAAAATCAGGACAAGGCGACGAAGCCGCAGACAGTACAAATAGTACGGAACCGATTCACTTGGTGCTTGAGCACCTTAGAGAATCGTTCTCTTTGAGCTAAGGCGAGGCAACGCCGTACCGGTTTTTGTTAATCCACTATAATCCGCCCGCATAAAATAGGATTGCCGCAAGCCGTGTTATACTGCGGCGTGTTTTACGGATTGTTCGGGCTATGGATTTATTATCGGTCTTCCACAAATACCGTCTGAAATATG
```

 

**tyrB �cyt5 (region TC)**

 

### N. cinerea ATCC14685

```
GCCGGACTGAATGCCTCGAATATCGCCTATGTTGCCGATGCATTTGCCGAAGTATTGAAATAAGAATTTGAAGTAAATCAAAAGGCCGTCTGAAAAATATTTATCTTTCAGACGGCCTCTTTATAATTGGCGCACCCAACAGGGATCGAACCTGTGACCTCCAGCTTCGGAAACTGACACTCTTCCAACTGAGCTATGGGTGCGGAAAACGTAAGATTAACGTAAATTCTGCAAATTGGCAAAGTTTATCTTGAGGCGTCAGGCTGATGCGCGGGGTATGAGGGATATTTGTTTGGGGATAAGCAAGTATTTTCAATGGATTATTTGCTTAAAAGAGACGCTGCCTGTGATTTTTTGCATATTTTCAATGATAGTGATTGGAAAGACCCTGTGAATTCAGTATAATCCAGCAAAATATTGTTAATTGCGTTTAACATACCAAACTATAATTTACAGCACAACCTAACCTGACGGCGAGGCCTACCAAATGAAACAACTCCGCGACAATAAAGCCCAAGGCTCTGCATTGTTCA
```

 

**N. lactamica ATCC23970**

CCGGGCTGAATACGTCGAATACCGGCTATGTTGCCCGCGCATTCGCGGAAGTCTTGAAATAAGAGTTTGAAGTAAGTCAAAAGGCCGTCTGAAAAGTATTTATCTTTCAGACGGCCTCTTTATAATTGGCGCACCCAACAGGGATCGAACCTGTGACCTCCAGCTTCGGAAACTGACACTCTTCCAACTGAGCTATGGGTGCGGAAAACGTAAGGTTAACGTAAATTCTGCAAATTGGCAAAGGTTATCTTGCGCCGGCAGGCGGCGTATGGCGGCGTGAGGGATGTTTCTTGGGAGATAATCCAGTATTTCAACGAATTATTTATTTTATATGGATACCGTTGGCAAATTTTTTCATATTTTCAATGATAGCGATTGGTAAGACCCTGTGAATTCAGTATNCANCCAGCAAAATATTGTTAACTGCGTTTAACACACCAAACTATAATTTACAGCACAACCTAACCTGACGGCGAGGCCTACCAAATGAAACAACTCCGCGACAATAAAGCCCAAGGCTCTGCACTGTTTA

 

### N. sicca ATCC9913

```
GCCGGACTGAATGCCTCGAATATCGCCTATGTCGCCGATGCGTTTGCCGAAGTGTCGAAATAAAACTCAAAAAACAAACAGATCGTCTGAAACCCGATGTAGTTTTATTTGAAGTAAATCAAAAGGCCGCCCGAAAAATTTTCATCTTTCAGACGGTCTCTCTATAATTGGCGCACCCAACAGGGATCGAACCTGTGACCTCCAGCTTCGGAAACTGACACTCTTCCAACTGAGCTATGGGTGCAGGGAAGGCGTAAGATTAACGCAAATCCCGCCTGTTGGCAAAGCCTATTATGCAGGATGGCGCAAACAAGACAAATTCGCAGAAAATTCCCCGCAAATAATCAACAACATAACCAATCCACCCCTTAACAAATGGCAAAAAACTGCCGCCTTTGCGTATATTTTCCACAAGAGCGATTGGAAAGCCCCTGTGAATTCAGTATAATCCAGCAAAATATTGTTAACTGCGTTTAACACACCAAACTATAACTCAAAGCACAACCTAACCTAACGGCGAGGCCTACCAAATGAAACAACTCCGCGACTCAAAAGCCCAAGGCTCTGCATTGTTCA
```
